# Expression and characterization of a *Talaromyces marneffei* active phospholipase B expressed in a *Pichia pastoris* expression system

**DOI:** 10.1038/emi.2016.119

**Published:** 2016-11-23

**Authors:** Yan He, Linghua Li, Fengyu Hu, Wanshan Chen, Huali Lei, Xiejie Chen, Weiping Cai, Xiaoping Tang

**Affiliations:** 1 Department of Infectious Diseases, Guangzhou Eighth People's Hospital, Guangzhou Medical University, Guangzhou 510060, Guangdong Province, China; 2 Research Institute of Infectious Diseases, Guangzhou Eighth People's Hospital, Guangzhou Medical University, Guangzhou 510060, Guangdong Province, China; 3 Department of Clinical Laboratory, Guangzhou Eighth People's Hospital, Guangzhou Medical University, Guangzhou 510060, Guangdong Province, China

**Keywords:** differential expression, heterologous expression, phospholipase B, *Talaromyces marneffei*, *Pichia pastoris*

## Abstract

Phospholipase B is a virulence factor for several clinically important pathogenic fungi, including *Candida albicans*, *Cryptococcus neoformans* and *Aspergillus fumigatus*, but its role in the thermally dimorphic fungus *Talaromyces marneffei* remains unclear. Here, we provide the first report of the expression of a novel phospholipase gene, designated *TmPlb1*, from *T. marneffei* in the eukaryotic expression system of *Pichia pastoris* GS115. Sensitive real-time quantitative reverse-transcription PCR (qRT-PCR) demonstrated that the expression of *TmPlb1* increased 1.85-fold in the yeast phase compared with the mycelial phase. *TmPlb1* contains an open reading frame (ORF) of 732 bp that encodes a protein of 243 amino acids. The conserved serine, aspartate and histidine catalytic triad and the G-X-S-X-G domain of TmPLB1 provide the structural basis for its molecular activity. The ORF of *TmPlb1* was successfully cloned into a pPIC9K vector containing an α-mating factor secretion signal that allowed the secretory expression of TmPLB1 in *P. pastoris*. The heterologous protein expression began 12 h after methanol induction and peaked at 96 h. Through analysis with SDS–polyacrylamide gel electrophoresis (SDS-PAGE), western blotting and mass spectrometry, we confirmed that TmPLB1 was successfully expressed. Through Ni-affinity chromatography, TmPLB1 was highly purified, and its concentration reached 240.4 mg/L of culture medium. With specific substrates, the phospholipase A1 and phospholipase A2 activities of TmPLB1 were calculated to be 5.96 and 1.59 U/mg, respectively. The high purity and activity of the TmPLB1 obtained here lay a solid foundation for further investigation.

## Introduction

*Talaromyces marneffei* (formerly *Penicillium marneffei*^1^) is a strictly thermally dimorphic fungus that can cause fatal systemic infections. The emerging HIV/AIDS pandemic in the 1980s, especially in Southeast Asia, including China, has resulted in the proliferation of opportunistic mycotic infections, often caused by *T. marneffei*, in HIV-infected individuals.^2^ Moreover, the past two decades have also witnessed a pronounced increase in the number of *T. marneffei* infections among non-HIV-infected patients with impaired cell-mediated immunity.^3^ *T. marneffei* can cause penicilliosis, whose common clinical manifestations include fever, weight loss, anemia, lymphadenopathy, hepatosplenomegaly, respiratory signs and skin lesions.^4^ In spite of the availability of antifungal therapy, the mortality of penicilliosis has reached 24.3% in China.^4^

*T. marneffei* grows as mycelia at 25 °C and transitions to yeast at 37 °C. The latter is considered to be the pathogenic phase, because yeast cells are capable of evading the host immune system.^5^ Despite its medical importance, the precise pathogenic mechanisms of *T. marneffei* are still poorly understood. Recognition and adherence to host cells,^6^ dimorphic phase transition^7^ and melanin secretion^8^ are thought to be pathogenic factors of *T. marneffei*. The secretion of enzymes, such as phospholipases, has also been proposed as a strategy used by parasites, bacteria and pathogenic fungi for invading the host and establishing infection.^9^

Phospholipases are a ubiquitous, heterogeneous group of enzymes that can hydrolyze one or more ester linkages of glycerophospholipids and can occasionally degrade the neutral lipids involved in membrane homeostasis, nutrient acquisition and the generation of bioactive molecules.^9^ They are also the key enzymes in pathogenic fungi that cleave host phospholipids, thereby resulting in membrane destabilization and host cell penetration.^10^ On the basis of their cleavage sites, phospholipases can be classified as phospholipase A (PLA), phospholipase B (PLB), phospholipase C (PLC) or phospholipase D (PLD).^11^ PLB can hydrolyze both the 1-acyl ester (PLA1) and the 2-acyl ester (PLA2) linkages of phospholipids and may also possess lysophospholipase activity.^9^ The role of PLB as a potential virulence factor for pathogenic fungi such as *Aspergillus fumigatus*,^12^ *Candida albicans*^13^ and *Cryptococcus neoformans*^14^ has been reported, although the underlying mechanism has yet to be elucidated. Whether the PLB of *T. marneffei* is involved in the pathogenesis has not been reported. By using RNA sequencing, we found that a PLB gene of *T. marneffe* shared a gene sequence with GenBank accession number PMAA_093290 of *T. marneffei* ATCC 18224 that was overexpressed in the yeast phase in comparative transcriptomic analysis (unpublished data). We expressed the protein encoded by this gene to provide clues as to its function.

The methylotrophic yeast *Pichia pastoris* is considered an excellent eukaryotic host for generating various recombinant heterologous proteins, owing to its easy manipulation and ability to achieve post-translational modification.^15^ The goal of this work was to clone and express the PLB gene, designated *TmPlb1*, from the yeast phase of *T. marneffei* in a *P. pastoris* yeast expression system. We also performed functional analysis of the *TmPlb1* gene and detected the differential gene expression in the life cycle of *T. marneffei.* These data establish a primary foundation for understanding the function of the *TmPlb1* gene in the pathogenesis of *T. marneffei*. To our knowledge, this is the first report on the heterologous eukaryotic expression of a phospholipase gene from the thermally dimorphic pathogenic fungus *T. marneffei.*

## Materials and methods

### Strains, media and growth conditions

*T. marneffei* strain GD-0079 was isolated from the bone marrow of an AIDS patient with the complication of penicilliosis. The strain was confirmed via bone marrow culture at Guangzhou Eighth People's Hospital and maintained at the Research Center for Pathogenic Fungi within the hospital. The GD-0079 isolate was inoculated on Sabouraud dextrose agar (Difco, BD, Baltimore, MD, USA) at 25 °C as mycelia and converted to yeast at 37 °C.

The *P. pastoris* expression kit containing pPIC9K vectors and *P. pastoris* strain GS115 were purchased from Invitrogen Corp. (Carlsbad, CA, USA). The *Escherichia coli* strain DH5α-competent cells were purchased from Takara (Dalian, China). *E. coli* cells with plasmids were cultured at 37 °C in Luria–Bertani medium (5 g/L yeast extract, 10 g/L tryptone,10 g/L NaCl and 15 g/L agar) containing 100 μg/mL ampicillin to maintain the plasmids. Minimal dextrose (MD) medium, yeast extract-peptone-dextrose (YPD) medium, buffered complex glycerol (BMGY) medium and buffered complex methanol (BMMY) medium were prepared according to the instructions of Invitrogen for *P. pastoris* fermentation.

### Total RNA and DNA isolation

The mycelia and yeast samples of *T. marneffei* were collected as previously described.^16^ After harvesting, all samples were stored at −80 °C or processed immediately for total RNA isolation. Approximately 100 mg of *T. marneffei* culture was pulverized under liquid nitrogen with a mortar and pestle. Extraction of the total RNA was carried out according to the manufacturer's instructions using the TRIzol reagent (Invitrogen) and treated with the RNase-free DNase I kit (Invitrogen) to eliminate DNA contamination. Yeast genomic DNA was extracted using a MasterPure Yeast DNA Purification Kit (Epicentre, Madison, WI, USA) according to the manufacturer's protocol.

### Bioinformatics and phylogenetic analysis of TmPLB1

The deduced amino acid sequences were analyzed with the Expert Protein Analysis System (http://expasy.org/tools/) and BLAST network service of the National Center for Biotechnology Information (NCBI). Open reading frame (ORF) prediction was performed using the ORF Finder Tool at the NCBI server (http://www.ncbi.nlm.nih.gov/projects/gorf/). The TmPLB1 protein sequences of *T. marneffei* and phospholipases from another 19 species were aligned using the Clustal X (http://www.clustal.org/), and a phylogenetic tree was constructed by the neighbor-joining method in MEGA version 6.0.^17^

### Quantitative real-time PCR

The RNA extracted from the mycelial and yeast phases was used as the template for real-time quantitative reverse-transcription polymerase chain reaction (qRT-PCR). Reverse transcription was performed using an MMLT-RT kit (Invitrogen, Shanghai, China). The qRT-PCR was performed on an Applied Biosystems ViiA 7 Real-Time PCR System (Applied Biosystems, Carlsbad, CA, USA) in 20 μL reaction mixtures containing PowerUp SYBR Green Master Mix (Applied Biosystems), 2 μL of complimentary DNA and 1 mM of gene-specific primers at 50 °C for 2 min and 95 °C for 2 min; this was followed by 40 cycles of 95 °C for 15 s and 60 °C for 1 min. Each amplification was followed by dissociation curve analysis to ensure that a single-size product was amplified. Three replicate reactions were carried out for each template and the NTC (no template control). The levels of *TmPlb1* mRNA were normalized to those of β-actin^18^ and the fold change was calculated with the 2^−ΔΔCT^ method.^19^ The specific primer pairs of TmPlb1 (qF1 and qR1) and reference gene β-actin (Act1F and Act1R) used for qRT-PCR analysis are shown in Table 1.

### Construction of *TmPlb1* expression vectors

The ORF of the *TmPlb1* gene from the yeast phase was amplified by RT-PCR with the following primer pairs: F1: 5′-CTA TAC GTA ATG TCG TAC CGG GCT CCC TTC G-3′ and R1: 5′-CAT GCG GCC GCT CAA TGA TGA TGA TGA TGA TGC AGA CCA GCT TCT TCG CCC G-3′ (the underlined sequences are a *Sna*BI site, a *Not*I site and a continuous hexa-His tag). The PCR amplification product was ligated into the pPIC9K vector between the *Sna*BI and *Not*I sites to yield the expression vector pPIC9k-*TmPlb1*. The recombinant transformants were identified by restriction digestion analysis.

### Transformation and screening for multicopy transformants

The expression vector pPIC9K-*TmPlb1* was linearized with *Sal*I and transformed into *P. pastoris* GS115 by electroporation with a Multiporator (Eppendorf, Hamburg, Germany) under the following conditions: 2 kV, 200 Ω and 25 μF. His^+^ transformants were screened on histidine deficient MD medium (0.4 μg/mL biotin, 1.34% yeast nitrogen base (without amino acids), 2% dextrose, 1.5% agar) for four days. The His^+^ colonies were then spotted onto YPD agar plates (1% yeast extract, 2% peptone, 2% dextrose, 2% agar) with 4.0 mg/mL of Geneticin418 to select multicopy transformants. The recombinant transformants were identified by PCR amplification and sequencing using the 5′ and 3′ alcohol oxidase 1 (AOX1) primers (Table 1).

### Protein expression in *P. pastoris*

A single colony of His^+^ transformant exhibiting resistance to 4.0 mg/mL Geneticin 418 was inoculated in 10 mL of YPD broth medium at 30 °C with constant shaking at 220 r.p.m. until an OD600 value of 2 was reached. The culture was then transferred to 500 mL BMGY medium (2% peptone, 1% yeast extract, 100 mM potassium phosphate, pH 6.0, 1.34% yeast nitrogen base (without amino acids), 0.4 μg/mL biotin and 1% glycerol) in a 2000 mL shaking flask. After 24 h of cultivation, the culture was centrifuged at 3000 × *g* for 5 min and resuspended in BMMY medium (2% peptone, 1% yeast extract, 100 mM potassium phosphate, pH 6.0, 1.34% yeast nitrogen base (without amino acids), 0.4 μg/mL biotin and 0.5% methanol). Then, 1% (v/v) methanol was added every 24 h to induce protein expression with constant shaking at 220 r.p.m. for 144 h. A transformant with empty pPIC9K plasmids was induced at the same time as a background control.

### SDS-PAGE analysis

One milliliter of the culture was centrifuged, and the supernatant was collected every 6 h. Next, 12% SDS–polyacrylamide gel electrophoresis (SDS-PAGE) was used to analyze the recombinant protein. The samples were mixed with NuPAGE LDS sample buffer (4 ×) (Life Technology, Carlsbad, CA, USA) and heated at 70 °C for 10 min before loading on the gel. The electrophoresis was performed at a constant voltage of 200 V for 35 min, and the gel was stained with Coomassie brilliant blue R-250.

### Purification with Ni-affinity chromatography

The culture was centrifuged, and the supernatant was loaded onto a 1 mL column that was pre-packed with Ni- nitrilotriacetic acid (Ni-NTA) Superflow resin from Qiagen (Hilden, Germany) and then washed with buffer A (50 mM NaH_2_PO_4_, 300 mM NaCl, 10 mM imidazole, 10% glycerol, pH 8.0) for 3 column volumes at 1 mL/min flow rate. Then, the protein was eluted with buffer B that, compared with buffer A, had a higher imidazole concentration of 200 mM. The fractions containing the protein were pooled and desalted by gradient dialysis. The desalted samples were resuspended in phosphate-buffered saline buffer with 10% glycerol and analyzed using SDS-PAGE, followed by western blotting.

### Western blot analysis

Proteins resolved by SDS-PAGE were electrotransferred to a polyvinylidene fluoride membrane by using the iBlot Dry Blotting System (Invitrogen). The membrane was blocked in 5% skim milk prepared in Tris-buffered saline containing 0.05% Tween-20 and then incubated with mouse-anti-His-Tag antibody (1:2000 dilution) (Qiagen) for 90 min at room temperature. After being washed, the membrane was incubated with horseradish peroxidase-conjugated horse anti-mouse IgG antibody (1:2000 dilution) (Asbio, Guangzhou, China) for 60 min at room temperature. The bound antibody was detected with chemoluminescent substrate (Asbio), and the signal was recorded with Tanon 4500 Imaging System (Tanon, Shanghai, China).

### Protein analysis and enzyme assays

Total protein concentrations were determined by the Pierce BCA assay kit (Thermo Scientific, Waltham, MA, USA) according to the manufacturer's instructions by using bovine serum albumin as a standard. The absorbance at 562 nm of the samples was measured with a spectrophotometer. The storage buffer of the purified protein (phosphate-buffered saline with 10% glycerol) was used as a background control.

Phospholipase enzymatic assays were performed using an EnzChek phospholipase A1 and A2 assay kit (Invitrogen). *N*-((6-(2,4-DNP) amino) hexanoyl)-1-(BODIPY FL C5)-2-hexyl-sn-glycero-3-phosphoethanolamine (PED-A1) and 1-*O*-(6-BODIPY 558/568-aminohexyl)-2-BODIPY FL C5-Sn-glycerol-3-phosphocholine (Red/Green BODIPY PC-A2 (Thermo Scientific, Waltham, MA, USA)) were used as selective substrates, and the fluorescence intensities were measured with a Spectra Max i3 fluorescence microplate reader (Molecular Devices, Sunnyvale, CA, USA). The storage buffer was also used as a background control run in parallel. All measurements were performed at least in triplicate.

### Mass spectrometry analysis

The protein band was excised from Coomassie-stained gels and analyzed by quadrupole-time of flight (Bruker Daltonics, Bremen, Germany) at the Beijing Honor Technology Co., Ltd (Beijing, China). Database searching was carried out using an in-house MASCOT server (Matrix Science, London, UK) to search NCBInr (ftp://ftp.ncbi.nih.gov/blast/db/). The search criteria were selected as follows: peptide tolerance±15 p.p.m., fixed modifications-carbamidomethyl (C), variable modifications-Gln-pyro-Glu (N-term Q) and oxidation (M), up to 1 missed tryptic cleavage allowed.

## Results

### Homology and phylogenetic analysis of TmPLB1

**Figure 1:**
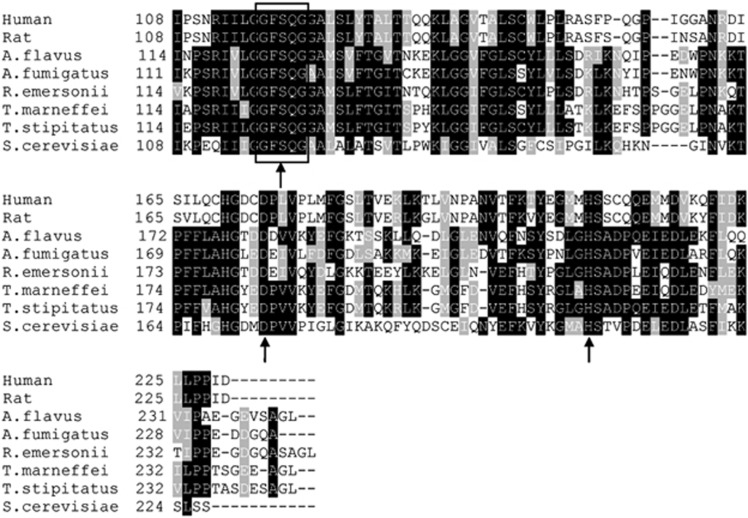
Sequence alignment of *TmPlb1* orthologs. Sequences were aligned using Clustal X and shaded by BOXSHADE. Shaded residues indicate ≥75% homology (black) or ≥50% homology (gray). The G-F-S-Q-G motif is boxed, and the catalytic triad sites are indicated by arrows. Abbreviations and accession numbers are as follows: human (*Homo sapiens*, GenBank: NP_006321); rat (*Rattus norvegicus*, GenBank: NP_037138); *A. flavus* (*Aspergillus flavus*, GenBank: XP_002380983); *A. fumigatus* (*Aspergillus fumigatus*, GenBank: KMK55316); *R. emersonii* (*Rasamsonia emersonii*, GenBank: XP_013325383); *T. stipitatus* (*Talaromyces stipitatus*, GenBank: XP_002485317); *S. cerevisiae* (*Saccharomyces cerevisiae*, GenBank: AAS56265).

The 732 bp ORF of *TmPlb1* encoded a protein of 243 amino acids without a signal peptide. The TmPLB1 protein had a predicted molecular mass of 26.89 kDa and a theoretical isoelectric point of 5.49. Multi-alignment analysis with Blastp and Clustal X indicated that TmPLB1 shared a high identity with phospholipase genes in other species, with the highest identity of 91% to *T. stipitatus*, 73% identity to *R. emersonii*, 67% identity to *A. flavus*, 66% identity to *A. fumigatus*, 40% identity to *S. cerevisiae* and 39% identity with both humans and rats (Figure 1). All of these proteins share a common G-F-S-Q-G motif and a putative catalytic triad of serine, aspartate and histidine (Figure 1).

**Figure 2:**
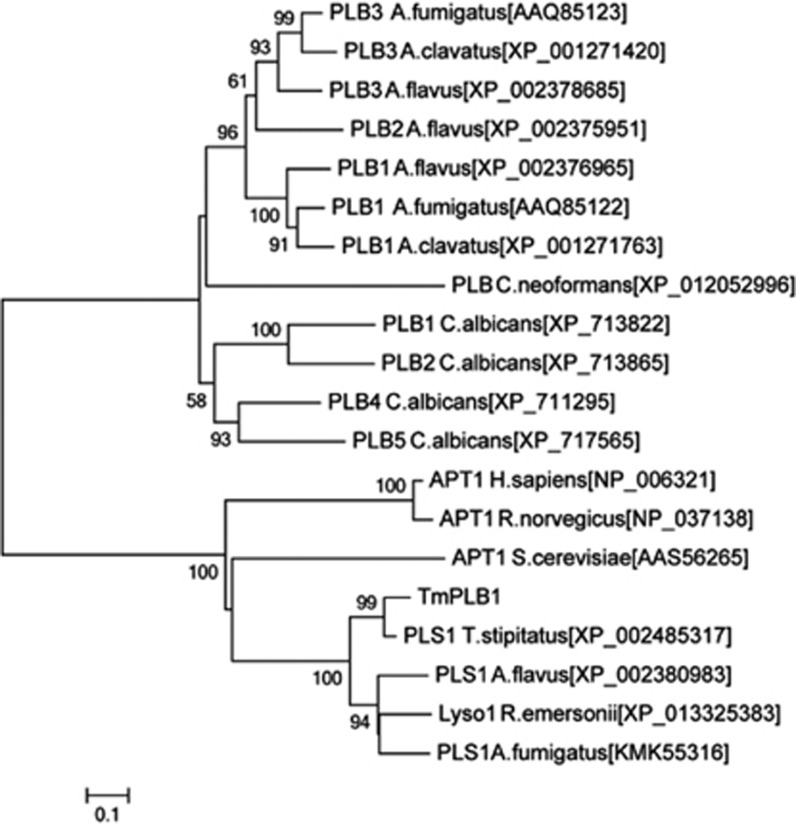
Phylogenetic analysis of PLB from various organisms. A neighbor-joining tree was constructed with MEGA version 6.0 using Poisson correction, and bootstrap values were calculated from 1000 trees. The scale bar indicates the estimated number of substitutions per 10 amino acids. Abbreviations: *Aspergillus fumigatus*, A. fumigatus; *Aspergillus clavatus*, A. clavatus; *Aspergillus flavus*, A. flavus; *Cryptococcus neoformans*, C. neoformans; *Candida albicans*, C. albicans; *Homo sapiens*, H. sapiens; *Rattus norvegicus*, R. norvegicus; *Saccharomyces cerevisiae*, S. cerevisiae; *Talaromyces stipitatus*, T. stipitatus; *Rasamsonia emersonii*, R. emersonii; phospholipase B, PLB; acyl-protein thioesterase 1, APT1; phospholipase 1, PLS1; lysophospholipase 1, Lyso1.

Phylogenetic analysis was carried out to identify the relationship between PLB from various organisms (Figure 2). Two groups were clearly generated. The first group comprised extracellular PLB from *Aspergillus* species and *Candida* species, and the second group contained various cytoplasmic phospholipases, including acyl-protein thioesterase 1 (APT1), that exhibited PLB and deacylation activity.^20^ Lacking a signal peptide, TmPLB1 was closer to cytoplasmic phospholipases than was secretory PLB.

### Differential expression of *TmPlb1* in mycelial and yeast cells

In this study, the differential expression of *TmPlb1* was analyzed by sensitive real-time RT-PCR. A melting curve showing a single peak at the melting temperature of the target nucleic acid sequence suggested specific amplification. Expression was determined as fold increased 2^−ΔΔCt^ levels relative to the phase with lower expression (mycelia). RT-PCR analysis revealed that the *TmPlb1* gene was expressed in both the mycelial and yeast phases, but a higher mRNA level was observed in the yeast phase (1.85-fold).

### Molecular cloning of *TmPlb1*

The ORF of the *TmPlb1* gene was cloned into the expression plasmid pPIC9K after the strong methanol-inducible AOX1 promoter and in-frame with the α-mating factor pre-propeptide for secretion. The gene was fused with a 6 × HIS tag at the C-terminus for easy detection and purification. After linearization with endonuclease *Sal*I, pPIC9K-*TmPlb1* was transformed into *P. pastoris* GS115-competent cells by electroporation. Genomic DNA was isolated from *P. pastoris* recombinants and controls, and PCR verification of the positive recombinants was then performed using 5′ and 3′ AOX1 sequencing primers (Table 1). For *P. pastoris* harboring pPIC9K-*TmPlb1*, a band corresponding to the size of the AOX1 gene plus the inserted *TmPlb1* gene (492+732 bp) was obtained (Supplementary Figure S1).

### Expression of the TmPLB1 protein and western blot analysis

**Figure 3:**
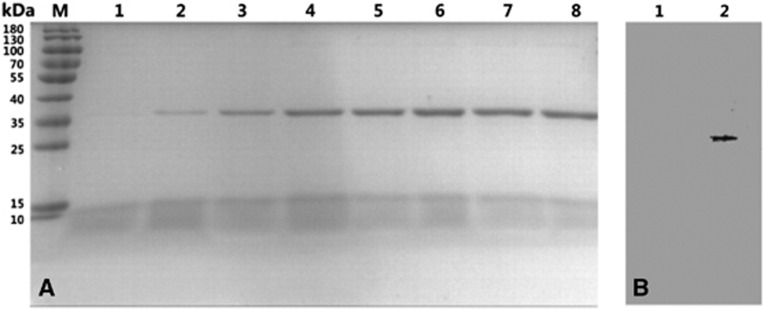
Expression of TmPLB1 in *P. pastoris* GS115. (**A**) SDS-PAGE analysis of TmPLB1 expressed in *P. pastoris* GS115 fermentation supernatants in shaking flask cultures. Lanes 1–8, *P. pastoris* induced with methanol for 6, 12, 24, 48, 72, 96, 120 and 144 h; M, standard protein marker (Fermentas, Burlington, ON, Canada). (**B**) Western blot analysis of TmPLB1. Lane 1, empty vector control; lane 2, TmPLB1 expression plasmids.

A selected multicopy transformant colony was cultured in a shaking flask to measure TmPLB1 expression. Fermentation broth was subjected to SDS-PAGE analysis. The protein expression began 12 h after methanol induction and peaked at 96 h. Only an obvious band at ∼35 kDa was detected in lanes 2–8 (Figure 3A). In the suspensions of an induced transformant harboring an empty pPIC9K plasmid, no obvious bands were detected (Supplementary Figure S2). The western blotting analysis results showed that an intense band was detected in the culture medium compared with the background control, thus clearly demonstrating that the protein was successfully expressed (Figure 3B).

### Purification of TmPLB1 by Ni-affinity chromatography and activity assay

**Figure 4:**
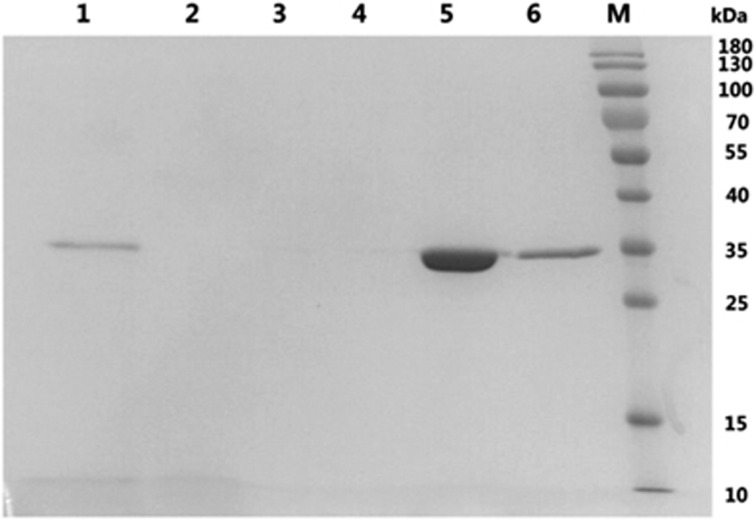
Purification of TmPLB1. Lane 1, *P. pastoris* GS115 fermentation supernatants induced with methanol for 96 h; lane 2, flow-through; lane 3, wash fractions 1; lane 4, wash fractions 2; lane 5, elution fractions 1; lane 6, elution fractions 2; M, protein molecular marker (kDa).

After 96 h of induction, the culture supernatant was collected and loaded onto Ni-NTA pre-packed columns, washed with buffer A containing low imidazole concentration and then eluted with a higher imidazole concentration buffer B. However, only a band at ∼35 kDa was observed (Figure 4). This result indicated that the *P. pastoris* GS115 recombinants secreted few proteins other than TmPLB1. We obtained 240.4 mg of purified TmPLB1 per L of culture supernatant. The specific PLA1 and PLA2 activity of the purified protein was 5.96 and 1.59 U/mg, respectively.

### Mass spectrometry analysis

The protein band was analyzed by quadrupole-time of flight mass spectrometry and was identified as the target protein with 651 independent peptide matches and a score of 19 147, and the protein sequence coverage reached 90% (*P*<0.05). The recombinant protein had a size ∼8 kDa larger than the predicted molecular mass owing to the presence of the C-terminal tag.

## Discussion

In pathogenic fungi, phospholipases are of considerable interest for their involvement in multiple cellular processes, including hyphal formation,^21^ mitotic growth^22^ and internalization into lung epithelial cells.^23^ However, the functions of phospholipases in *T. marneffei* remain unclear. In this report, we systematically analyzed a PLB gene from *T. marneffei* and provide a primary foundation for further investigating the functions of PLB and its role in the pathogenic mechanisms of *T. marneffei*.

The life cycle of the thermally dimorphic fungus *T. marneffei* can be divided into a multicellular, filamentous vegetative form and a unicellular yeast-like phenotype.^24^ Our previous comparative transcriptomic analysis (unpublished data) has indicated that the *TmPlb1* gene is overexpressed in the yeast phase (1.60-fold). In this study, sensitive qRT-PCR was used to analyze the differential expression of *TmPlb1* between the mycelial phase and yeast phase, and the results confirmed that the mRNA level of *TmPlb1* increased in the pathogenic yeast phase (1.85-fold). This finding is consistent with the results of a comparative transcriptomic analysis and suggests that the *TmPlb1* gene may play a role in the pathogenesis of *T. marneffei.* To understand the specific functions of *TmPlb1*, targeted gene disruption should be carried out in the future.

The deduced TmPLB1 protein contained the conserved catalytic triad of serine, aspartate and histidine^25^ and the G-F-S-Q-G sequence corresponding to the catalytic domain consensus sequence (G-X-S-X-G) in phospholipases^26^ (Figure 1). The catalytic triad and domain together provide a crucial structural basis for the molecular activity of TmPLB1. The evolution of the *TmPlb1* gene in *T. marneffei* has paralleled the evolution of the fungus. TmPLB1 clusters into the intracellular phospholipase group and more phylogenetically closer to mitosporic *Trichocomaceae*, including other *Penicillium* species and *Aspergillus* species (Figure 2). We hypothesize that the intracellular PLB gene cluster was acquired by the common ancestor of this group of fungi and that subsequent gene rearrangement and divergence have resulted in the various gene orders of individual genes in different fungi.^27^

To date, no active phospholipase of *T. marneffei* has been expressed in either a prokaryotic or eukaryotic expression system. Compared with prokaryotic expression systems, eukaryotic expression systems are more likely to properly process, fold and chemically modify foreign proteins.^28^ The methylotrophic yeast *P. pastoris* is easy to manipulate and grows rapidly on inexpensive media at high densities. Therefore, we selected the eukaryotic expression system of *P. pastoris* to express the TmPLB1 protein. The ORF of the *TmPlb1* gene was successfully cloned into the pPIC9K vector that contained an α-mating factor secretion signal and allowed the secretory expression of TmPLB1 in *P. pastoris* GS115. Expression of enzymatically active TmPLB1 was induced with methanol. We found that 96 h after methanol induction was the optimal collection time for TmPLB1 (Figure 3A) and that further extending the induction time may reduce the yield of TmPLB1 because of an inadequate oxygen supply for the accumulating *P. pastoris* GS115.^15^ *P. pastoris* secretes only very low levels of endogenous protein into the growth medium; thus, the secreted TmPLB1 protein constituted the majority of the total protein in the medium (Figure 3A); this advantage allows for the purification of the expressed protein.^29^ Ni-affinity chromatography was used to capture TmPLB1 and allow contaminants to be washed away (Figure 4). After gradient dialysis, the final concentration of highly purified TmPLB1 reached 240.4 mg/L of culture supernatant, and the PLA1 and PLA2 activities of TmPLB1 were calculated to be 5.96 and 1.59 U/mg, respectively, an amount sufficient for subsequent functional studies.

SDS-PAGE analysis indicated that the secreted protein had an apparent molecular weight of ∼35 kDa, and this was ∼8 kDa larger than the predicted molecular mass. After western blotting (Figure 3B) and quadrupole-time of flight mass spectrometry analyses, we confirmed that the secreted protein was TmPLB1. The positively charged C-terminal 6 × HIS tag probably slowed the migration rate of TmPLB1 during SDS-PAGE and resulted in the greater apparent molecular weight.

Our results demonstrate that high-purity TmPLB1 protein can be abundantly produced by *P. pastoris* GS115. After purification and identification of TmPLB1, we observed PLA1 and PLA2 activities *in vitro*, thus suggesting that TmPLB1 might play an active role in *T. marneffei.* Although TmPLB1, as an intracellular phospholipase, is unlikely to be involved in the direct invasion of host cells, the successful expression of TmPLB1 in *P. pastoris* lays a solid foundation for its future investigation.

## Figures and Tables

**Table 1:** Sequences of primers used in this study

*Primer*	*Sequence*	*Length (bp)*
qF1	5′-ATCGTCCTACCTGTTACTT-3′	148
qR1	5′-ATGCTTCTGCGTCATATC-3′	148
Act1F	5′-TGATGAGGCACAGTCTAAGC-3′	155
Act1R	5′-CTTCTCTCTGTTGGACTTGG-3′	155
5′AOX1	5′-GACTGGTTCCAATTGACAAGC-3′	492
3′ AOX1	5′-GCAAATGGCATTCTGACATCC-3′	492
