# Supplementary material for: Expression and characterization of a Talaromyces marneffei active phospholipase B expressed in a Pichia pastoris expression system
Source: Emerg Microbes Infect. 2016 Nov 23;5(11):e120–. doi: 10.1038/emi.2016.119 (PMC5148023; doi:10.1038/emi.2016.119)
Supplement: Supplementary Figure S1 [file emi2016119x1.pdf]

A B

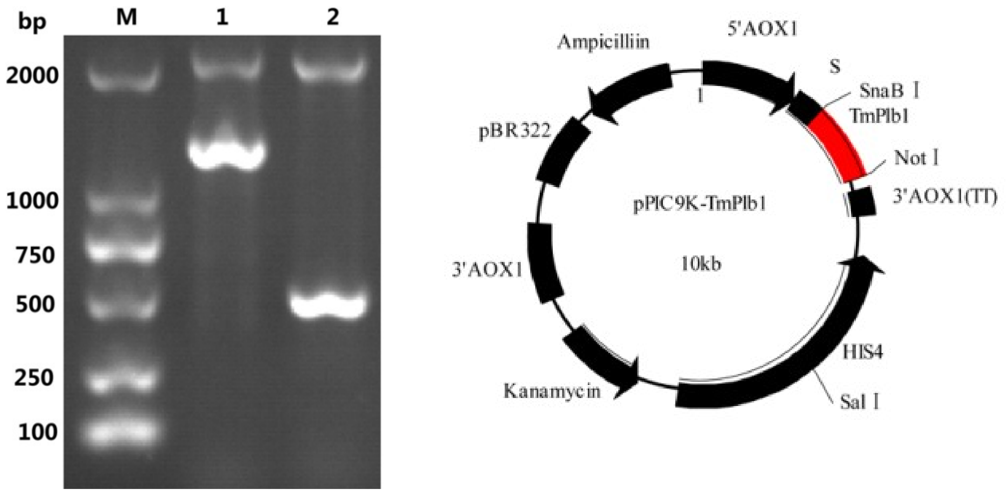

Supplementary Figure S1 (**A**) Transformation of pPIC9K-*TmPlb1* into *P. pastoris* GS115 competent cells. M, DNA Marker; 1, PCR with 5’ and 3’ AOX1 primers using pPIC9K-*TmPlb-GS115* as template; 2, PCR with 5’ and 3’ AOX1 primers using pPIC9K-*GS115* as template. (**B**) The schematic diagrams of pPIC9K-*TmPlb1*.
