# Supplementary material for: Expression and characterization of a Talaromyces marneffei active phospholipase B expressed in a Pichia pastoris expression system
Source: Emerg Microbes Infect. 2016 Nov 23;5(11):e120–. doi: 10.1038/emi.2016.119 (PMC5148023; doi:10.1038/emi.2016.119)
Supplement: Supplementary Figure S2 [file emi2016119x2.pdf]

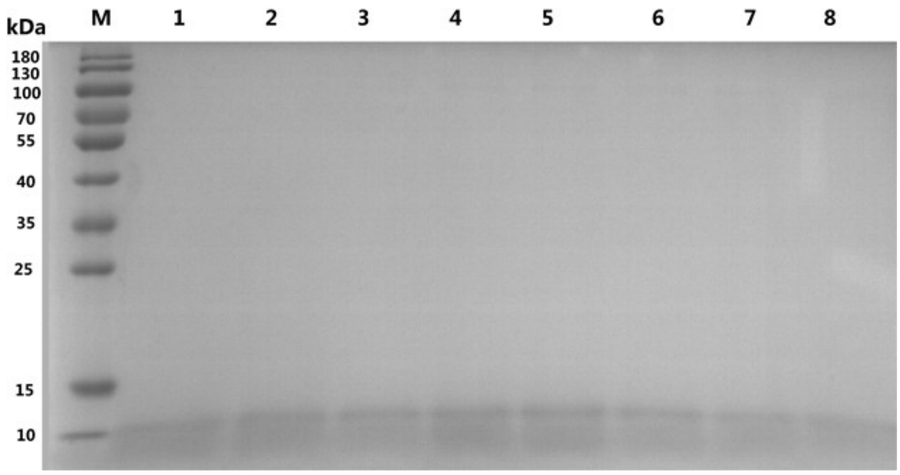

Supplementary Figure S2 Protein expression in *P. pastoris* GS115 with empty pPIC9K plasmids. M, standard protein marker (Fermentas); lanes 1–8, *P. pastoris* GS115 induced with methanol for 6, 12, 24, 48, 72, 96, 120 and 144 h.
